# Molecular epidemiology and genomic analysis of bulbul coronavirus in Guangdong, China

**DOI:** 10.3389/fvets.2026.1659863

**Published:** 2026-03-04

**Authors:** Lina Zhang, Shuting Chen, Huihua Li, Lixia Li, Hao Liu

**Affiliations:** 1 Eco-Engineering Department, Guangdong Eco-Engineering Polytechnic, Guangzhou, China; 2 School of Animal Science and Technology, Foshan University, Foshan, China

**Keywords:** bulbul coronavirus, genetic evolution, metagenomic sequencing, real-time PCR, sequence analysis

## Abstract

**Introduction:**

Bulbul coronavirus (BuCoV), a delta coronavirus recently identified in passerine birds, remains poorly characterized regarding its ecology and evolutionary dynamics. This study aimed to determine the prevalence of BuCoV in wild avifauna, clarify its evolutionary relationship with other delta coronaviruses, and identify genetic signatures potentially relevant to host adaptation and cross-species transmission in southern China.

**Methods:**

From 2023 to 2024, we conducted molecular surveillance across 12 regions in Guangdong Province, China. A total of 2,145 avian fecal samples were collected and screened for BuCoV using real-time quantitative PCR. The complete genomes of representative strains were obtained using next-generation sequencing. Subsequent analyses included phylogenetic reconstruction using maximum likelihood methods, recombination detection using RDP4 and SimPlot, and comparative amino acid analysis.

**Results:**

BuCoV was detected exclusively in Shenzhen (3/168, 1.78%), with all positive samples originating from bulbuls (Pycnonotus spp.). The Shenzhen strain GD2411 exhibited the highest nucleotide identity with BuCoV strains HKU11-796 (97.26%) and HKU11-934 (96.79%), but far lower similarity (78.9%–82.4%) to other delta coronaviruses. Phylogenetic analysis placed GD2411 in a monophyletic clade with HKU11 strains. Recombination analyses revealed mosaic structures within the spike (S) gene, involving multiple coronavirus lineages. Thirty-one amino acid substitutions were detected in the S protein, together with mutations in RdRp, 3CLpro, and nucleocapsid.

**Discussion:**

These findings suggest that BuCoV GD2411 emerged through inter-lineage recombination and is undergoing adaptive evolution, particularly in the spike protein. The detection of BuCoV exclusively in Shenzhen, a critical node in the East Asian-Australasian Flyway, suggests that migratory birds may facilitate viral dissemination. The identified mutations may affect viral replication, host adaptation, or immune evasion. These findings provide essential baseline genomic and epidemiological data critical for understanding BuCoV diversity and assessing potential zoonotic risks in southern China.

## 1 Introduction

Coronaviruses (CoVs) are enveloped, positive-sense single-stranded RNA viruses belonging to the family Coronaviridae within the order Nidovirales. They infect a wide range of hosts, including mammals and birds, and are classified into four genera: Alpha coronavirus, Beta coronavirus, Gamma coronavirus, and delta coronavirus. The White-eyed Bulbul (*Pycnonotus sinensis*), widely distributed in the Yangtze River basin and southern China, serves as an important biological control agent in agricultural and forest ecosystems owing to its extensive consumption of insect pests. BuCoV, first identified in Hong Kong in 2009, was taxonomically assigned to the genus Delta coronavirus (DeltaCoV), primarily infects White-eyed Bulbuls and other avian species. Infection may manifest as clinical signs involving the intestinal, respiratory, and reproductive systems, posing potential threats to avian health (1). Despite BuCoV’s demonstrated pathogenicity in birds, research on this virus remains limited both domestically and internationally, leaving critical knowledge gaps regarding its epidemiology and zoonotic potential. The ongoing SARS-CoV-2 pandemic has dramatically underscored the imperative for proactive surveillance of coronavirus diversity in wildlife reservoirs, particularly given the demonstrated capacity for cross-species transmission among delta coronaviruses (1–4). Notably, these avian coronaviruses exhibit unique RNA-dependent RNA polymerase template-switching capabilities during replication, driving frequent recombination events (5–7). In this study, we conducted large-scale molecular surveillance of wild birds across Guangdong from 2023 to 2024, followed by metagenomic sequencing and genomic characterization of representative BuCoV strains. By integrating phylogenetic, recombination, and mutational analyses, we aimed to determine the prevalence of BuCoV in wild avifauna, clarify its evolutionary relationship with other delta coronaviruses, and identify genetic signatures potentially relevant to host adaptation and cross-species transmission.

## 2 Materials and methods

### 2.1 Sample collection

A spatially stratified sampling design was implemented across major avian habitats in Guangdong Province. Fresh fecal samples were collected from March 2023 to November 2024. Specimens were immediately preserved in viral transport medium (Hank’s Balanced Salt Solution supplemented with 100 U/mL penicillin, 100 μg/mL streptomycin, and 2.5 μg/mL amphotericin B) and cryopreserved at −80 °C within 24 h to ensure RNA integrity.

### 2.2 Virus detection

#### 2.2.1 RNA extraction and reverse transcription

For each avian fecal sample, total RNA was extracted from 200 μL of supernatant using a commercial extraction kit (Fast Pure® Viral DNA/RNA Mini Kit, Vazyme Biotech Co., Ltd., China). The extracted RNA was subsequently reverse transcribed into complementary DNA (cDNA) using a reverse transcription kit (HiScript® II 1st Strand cDNA Synthesis Kit, Vazyme Biotech Co., Ltd.). The synthesized cDNA was stored at −20 °C pending further analysis.

#### 2.2.2 Real-time quantitative PCR detection

Based on available BuCoV gene sequences in GenBank, specific qPCR primers were designed using Primer Premier 5 software and synthesized by Sangon Biotech (Shanghai) Co., Ltd. The primer pair (YG-BuCoV-F1: 5’-ACCGAGCCTAGTACGGATAA-3′; YG-BuCoV-R1: 5’-GCAAAGTGATTCGGTGAGATAAG-3′) yielded a 113-bp amplicon. The qPCR reaction was performed in 20-μL volumes containing: 10 μL of 2 × SGE, 1.0 μL each of YG-BuCoV-F1 and YG-BuCoV-R1 primers, 4.0 μL of template cDNA, and 4.0 μL of ddH_2_O. Thermal cycling conditions comprised: initial denaturation at 95 °C for 3 min; 40 cycles of denaturation at 95 °C for 5 s and annealing/extension at 60 °C for 20 s. The specificity of the qPCR assay was validated by melting curve analysis.

### 2.3 Whole genome sequencing

Viral metagenomic detection was performed using the HiPure Viral RNA Kit (Magen) for viral RNA extraction. Following extraction, the VAHTS Universal V10 RNA-seq Library Prep Kit for Illumina (Novogene) was employed for library construction. High-throughput sequencing was performed on the Illumina HiSeq 2,500 platform to obtain viral genome sequence information. After sequencing, Raw sequencing reads were initially processed for quality control and filtering using the fastp software to enhance the accuracy and reliability of the data. Subsequently, the MEGAHIT assembler was utilized to assemble the filtered reads into complete viral genome sequences.

### 2.4 Homology and phylogenetic evolutionary analysis

Homology analysis of genomic sequences from 29 publicly available coronavirus strains (retrieved from GenBank) was performed using DNAStar software. This analysis encompassed key viral proteins-including ORF1ab (containing 3CLpro, and RdRp domains), nucleocapsid (N), and spike (S) proteins-for both nucleotide and amino acid sequence comparisons. Additionally, potential recombination events in the S protein gene of the GD2411 strain were investigated using RDP4 software. Phylogenetic analysis was conducted using MEGA 11 software, employing the Maximum Likelihood (ML) method with 1,000 bootstrap replicates under the Kimura 2-parameter model to infer evolutionary relationships (8).

## 3 Results

### 3.1 Detection of BuCoV in wild birds

Among the 2,145 fecal samples collected from 12 regions in Guangdong Province (Supplementary Figure S1), BuCoV RNA was detected exclusively in Shenzhen. Of the 168 samples obtained from this site, three tested positive, yielding a prevalence rate of 1.78% (3/168). Melting curve analysis demonstrated a single sharp peak at 80.8 °C for all positive samples, with no primer-dimer artifacts observed in negative controls (Supplementary Figure S2). No BuCoV-positive samples were identified in the remaining regions. The three positive samples originated from bulbuls (*Pycnonotus* spp.), confirming their role as the natural host reservoir in this region (Table 1).

**Table 1:** Collection of bird fecal samples from 12 bird habitats in Guangdong Province.

Region	Habitat type	Number of samples	BuCoV positive samples
Guangzhou	Haizhu wetland	93	0
	Nansha wetland	90	0
Shenzhen	Shenzhen mangrove reserve	168	3
Huizhou	Elephant Head Mountain Reserve/West Lake Park	66	0
Shanwei	Haifeng Nature Reserve	144	0
Jieyang	Great Beishan Forest Park/Binjiang Park	90	0
Shantou	South Australia Island Reserve	87	0
Chaozhou	Western Australia egret Paradise	149	0
Zhanjiang	Taurus Island mangrove forest	89	0
Maoming	Shuidong Bay Park	131	0
Yangjiang	Yuanyang Lake Park	92	0
Jiangmen	Xinhui Rock Creek Park	60	0
Foshan	Foshan Xianxi Reservoir	886	0
Total		2,145	3

### 3.2 Genomic features of BuCoV GD2411

High-throughput sequencing successfully recovered near-complete genomes from the three positive samples. One representative strain, designated GD2411, was selected for detailed analysis owing to its superior coverage and completeness. The genome of GD2411 comprised 26,482 nucleotides, with a typical delta coronavirus genome organization: 5′-ORF1ab-S-E-M-N-3′ (7). ORF1ab (18,791 bp) encodes a 6,262-amino acid polyprotein harboring PLpro, 3CLpro, RdRp, and other nonstructural proteins. Structural genes include the S gene (nt 19,382-22,876, 3,495 bp) and N gene (nt 24,121–25,171, 1,051 bp). Moreover, GD2411 retains the conserved Delta CoV transcriptional regulatory sequence 5′-ACACCA-3′, confirming its taxonomic placement within the genus delta coronavirus (7).

### 3.3 Homology and phylogenetic relationships

**Figure 1:**
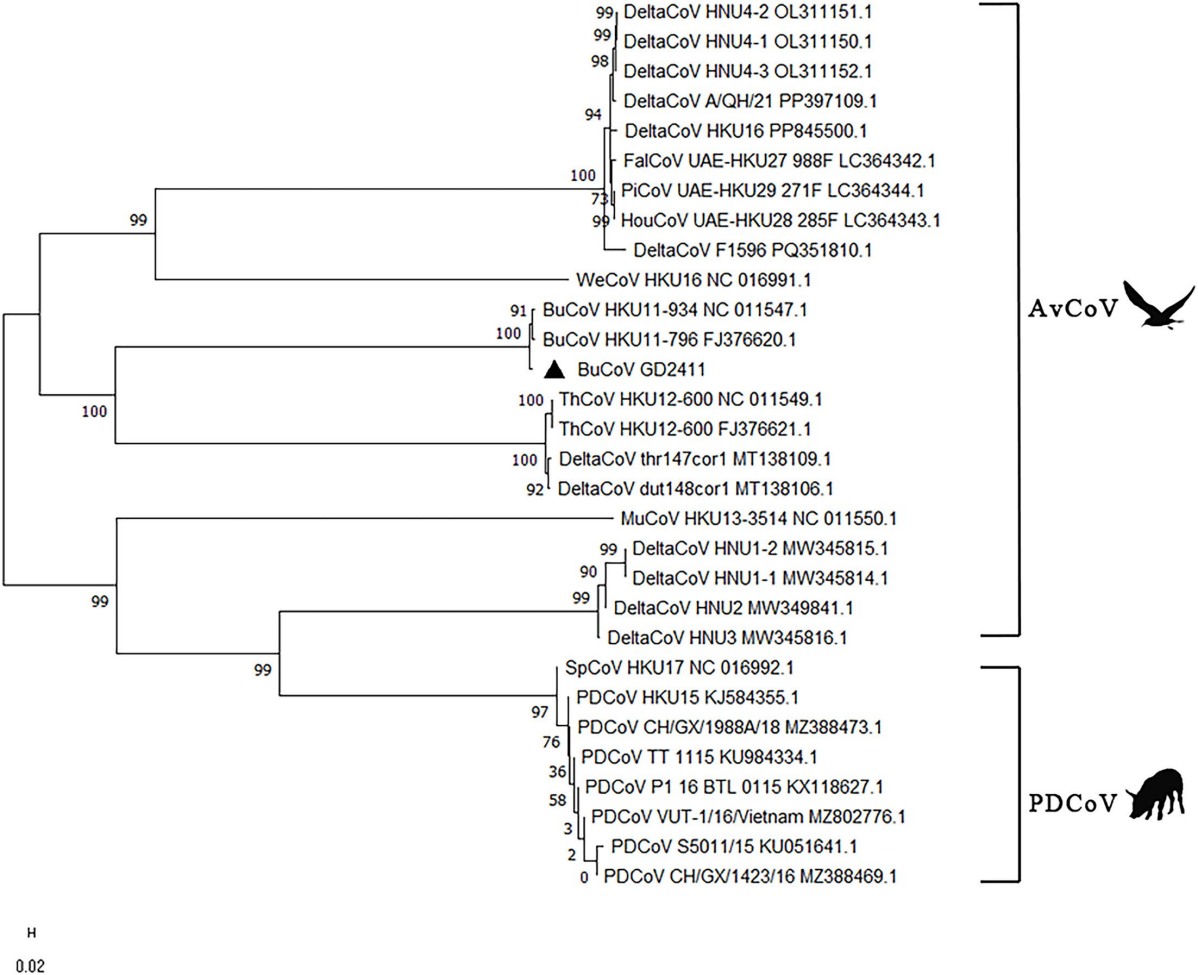
The phylogenetic tree was constructed using the complete genome sequence of BuCoV GD2411 and 29 other coronavirus strains from various animal hosts in MEGA 11 software. Bootstrap analysis with 1,000 replicates was performed to assess the robustness of the tree topology. Bootstrap values are indicated at each node of the tree. The triangle symbol represents the isolate BuCoV GD2411.

Pairwise sequence comparisons revealed that GD2411 shared the highest nucleotide identity with BuCoV strains HKU11-796 (97.26%) and HKU11-934 (96.79%). In contrast, nucleotide similarity with other delta coronaviruses-including porcine delta coronavirus (PDCoV), sparrow coronavirus HKU17, and night heron coronavirus HKU19-was substantially lower (78.9%–82.4%). Maximum-likelihood phylogenetic analysis based on complete genomes clustered GD2411 within a monophyletic clade together with HKU11 strains, distinctly separated from other delta coronaviruses (Figure 1). Furthermore, whole-genome comparison revealed that GD2411 shared 81.30–81.38% nucleotide identity with other delta coronavirus strains, including thr147cor1 (MT138109, 81.38%), dut148cor1 (MT138106, 81.35%), and ThCoV HKU12 (FJ376621, 81.30%), while identity with porcine delta coronavirus (PDCoV) strains ranged from 78.9% to 82.4% (Table 2) (Supplementary Figure S3).

**Table 2:** Nucleotide homology analysis of the ORF1ab gene among BuCoV GD2411 and other delta coronaviruses.

Protein	HKU11-796/HKU11-934 identity (%)	DeltaCoV thr147cor1/dut148cor1/HKU12-600 identity (%)
3CLpro	97.7–98.8	78.6–79.2
RdRp	96.9–97.9	83.8–84.0
Hel	98.1–98.9	87.3–87.7
S	92.3–94.5	51.5–52.2
N	98.2–98.6	73.7–73.8

### 3.4 Gene-specific homology and evolutionary divergence

**Figure 2:**
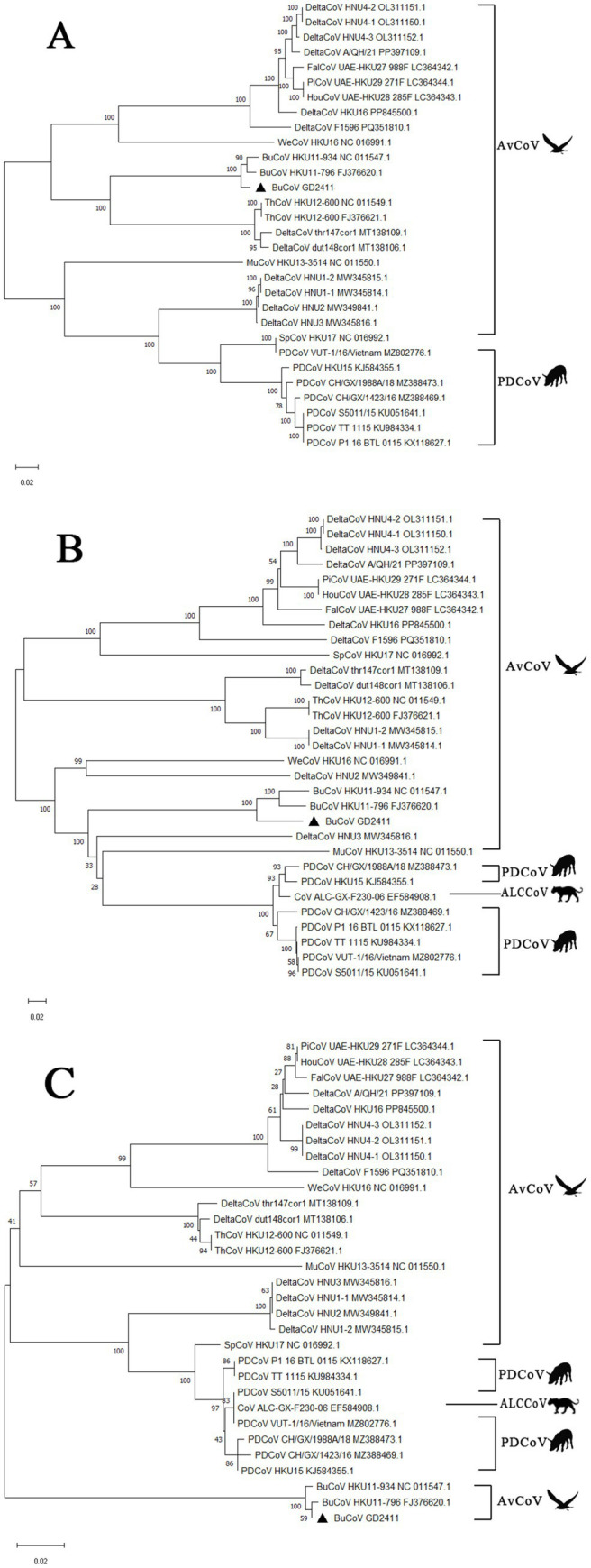
Phylogenetic trees were constructed using the ORF1ab, S, and N gene sequences of BuCoV GD2411 and other coronaviruses in MEGA 11 software. The analysis employed the Bootstrap method with 1,000 replicates to assess the reliability of the phylogenetic trees. The numerical values at each node of the tree represent Bootstrap values, which indicate the statistical support for the corresponding branches. Triangular symbols denote the positions of different proteins of BuCoV GD2411 in the trees. **(A)** Shows the phylogenetic tree of ORF1ab. **(B)** Represents the phylogenetic tree of the S protein. **(C)** Illustrates the phylogenetic tree of the N protein.

Consistent with whole-genome findings, ORF1ab and its encoded domains (3CLpro, RdRp, and Hel) as well as the N protein exhibited the highest similarity with HKU11-796 and HKU11-934 (99.4% and 99.1%, respectively), with markedly reduced identity (75.6%–83.7%) compared to other delta coronaviruses (Table 2; Figures 2A, 2C). In contrast, the spike (S) protein gene demonstrated greater divergence, displaying the highest identity to HKU11-796 (94.5%) and HKU11-934 (92.3%), yet notably higher similarity to porcine delta coronavirus (PDCoV; 66.8%–67.2%) than to other avian strains (51.6%–66.0%) (Figure 2B). This pattern suggests distinct evolutionary trajectories for the S gene (Supplementary Figure S3).

### 3.5 Recombination events in the spike gene

**Figure 3:**
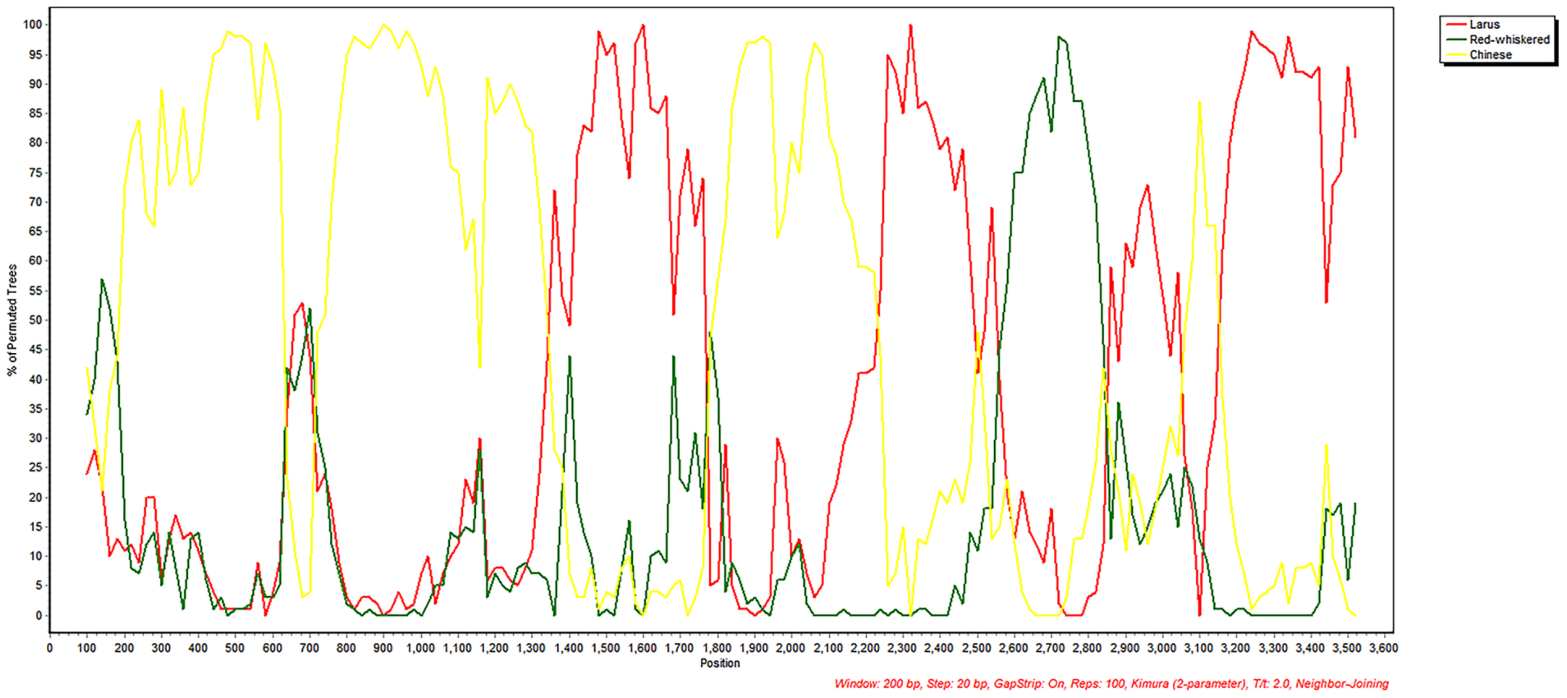
The figure illustrates the genetic recombination events involving two BuCoV strains, HKU11-796 and HKU11-934, as well as the avian coronavirus Fiocruz-F1596.

Recombination analysis using RDP4 and SimPlot identified significant recombination breakpoints within the S gene of GD2411. These recombination events likely originated from homologous recombination among HKU11-796, HKU11-934, and the avian coronavirus Fiocruz-F1596, supporting the critical role of recombination in the evolution and diversification of BuCoV (Figure 3).

### 3.6 Amino acid substitutions

Comparative amino acid analysis, based on the aligned translation frames of viral open reading frames, identified 37 substitutions in GD2411 relative to HKU11-796/HKU11-934. As illustrated in the schematic translation map, the majority of mutations were localized within the S protein (*n* = 31), with 18 mutation sites densely clustered within a 92-bp region (nt 431–523), including V433I, L453D, D489N, T512E, and Y518N. Additional substitutions were identified in RdRp (*n* = 4: V3764I, D3776G, S3836H, and D4352N), 3CLpro (*n* = 1: S2742N), and the N protein (*n* = 1: S154A) (Table 3) (Supplementary Figure S4). These mutations may potentially influence viral replication efficiency, host receptor binding affinity, and immune evasion mechanisms.

**Table 3:** Comparison of amino acid mutation sites.

Reference strains		Amino acid position										
		RdRp				3Clpro	N	S				
		3,764	3,376	3,836	4,352	2,742	154	433	453	489	512	518
GenBank number	Name	V	D	S	D	S	S	V	L	D	T	Y
PV544238	GD2411	I	G	H	N	N	A	I	D	N	E	N
FJ376620	HKU11-796		E	N			A		H		S	R
NC011547	HKU11-934		E	N					H		S	R
MT138106	dut148cor1					N	P	S	P		I	K
MT138109	thr147cor1					N	P	S	P		I	K
PP397109	A/QH/21		A	P				H		R		
PP845500	HKU16		A	P				Y		R		
OL311150	HNU4-1		A	P				H		R		
OL311151	HNU4-2		A	P				H		R		
OL311152	HNU4-3		A	P				H		R		
MW345814	HNU1-1					D		T	P		I	S
MW345815	HNU1-2					D		T	P		I	S
MW345816	HNU3					D			S	E	D	H
MW349841	HNU2					D			K	S	Y	T
LC364343	UAE-HKU28 285F		A	P				Y		R		
LC364344	UAE-HKU29 271F		A	P				Y		R		
LC364342	UAE-HKU27 988F		A	P			P	H		R	A	
FJ376621	HKU12-600					N	P	S	P		I	S
NC011549	HKU12-600					N		S	P		I	S
PQ351810	F1596		A	P				Q		K		
KJ584355	HKU15					D		I	P	E	E	T
KU051641	S5011/15					D		I	P	E	E	T
MZ388469	CH/GX/1423/16					D		I	P	E	E	T
MZ388473	CH/GX/1988A/18					D		I	P	E	E	T
NC016992	HKU17					D		Y	K	E		
MZ802776	VUT-1/16/Vietnam					D		I	P	E	E	T
NC016991	HKU16			I	N	N		H		S	Y	N
NC011550	HKU13-3514		Q	P		D		L	S	V	L	R

## 4 Discussion

The detection of BuCoV exclusively in Shenzhen Mangrove Nature Reserve (1.78% positivity) represents a significant geographic extension of this virus from Hong Kong to the southern coastal region of mainland China (1). Shenzhen Mangrove Reserve constitutes a critical node in the East Asian-Australasian Flyway, serving as a wintering ground for migratory birds from Southeast Asia and northern China, where avian species from Siberia, Australia, and Southeast Asia converge during wintering periods (9). The high genetic similarity (97.26%) between GD2411 and Hong Kong strains HKU11-796/934 strongly suggests that migratory birds function as vectors, facilitating viral introduction to this specific locality.

The focal distribution of BuCoV, detected exclusively in Shenzhen despite sampling across 12 regions throughout Guangdong Province, warrants careful consideration. Several factors may account for this spatially restricted pattern. First, the Shenzhen Mangrove Nature Reserve represents a unique ecological niche characterized by high biodiversity, dense vegetation coverage, and year-round warm and humid conditions (average temperature 25 °C, relative humidity >80%). These environmental conditions may favor coronavirus persistence and transmission among avian hosts. In contrast, other sampled habitats, including inland wetlands (Haizhu, Nansha), mountain reserves (Elephant Head Mountain), and freshwater parks, present different microclimatic conditions that may be less conducive to BuCoV survival outside hosts.

Second, field observations during sampling revealed that Shenzhen Mangrove Reserve hosts the highest density of white-eyed bulbuls (*Pycnonotus sinensis*) in Guangdong Province. The local bulbul population comprises year-round residents, facilitating continuous virus circulation. Higher host density promotes increased contact rates and more efficient viral transmission, potentially sustaining endemic circulation that may not be achievable in areas with lower population densities. In contrast, other regions exhibited lower host densities and higher proportions of transient individuals, thereby reducing transmission opportunities.

Whole-genome sequencing and phylogenetic analyses demonstrated that BuCoV GD2411 shares the highest nucleotide identity (97.26% and 96.79%, respectively) and closest evolutionary relationship with strains HKU11-796 and HKU11-934. Furthermore, GD2411 exhibited 81.30%–81.38% nucleotide identity with Delta CoV strains including thr147cor1, dut148cor1, and ThCoV HKU12 (Table 2), with which it clustered within the same clade, indicating its classification as a novel Delta CoV member. Phylogenetic analysis of GD2411-specific genes, including 3CLpro, RdRp, and Hel within ORF1ab, as well as N genes-corroborated the whole-genome results, confirming its close genetic linkage to HKU11-796/HKU11-934. The spike (S) gene of GD2411 showed the highest identity with HKU11-796 (94.5%) and HKU11-934 (92.3%), but markedly lower identity (66.8%–67.2%) with other DeltaCoV members (e.g., PDCoV strains MZ388473, MZ388469, KU051641, KX118627, KU984334, MZ802776, and KJ584355) and Asian leopard cat coronavirus (ALC/GX/F230/06; 68.8%). This pronounced divergence suggests host-driven differential selection pressure on the S protein during coronavirus evolution, resulting in significant intra-genus divergence.

Recombination represents a hallmark of coronavirus evolution, particularly in the spike gene, which mediates host entry and tropism (10–12). GD2411 emerged through inter-lineage recombination involving HKU11-796, HKU11-934, and the avian coronavirus Fiocruz-F1596. This mosaic structure of the S gene likely confers altered antigenic or receptor-binding characteristics, potentially expanding BuCoV’s host range, which serves as a key driver of cross-species transmission, particularly through S gene recombination (13–19). These recombination events have been increasingly reported in avian delta coronaviruses and are considered a principal mechanism underlying cross-species transmission. The identification of a viable recombinant strain in a wild bird population underscores the importance of active surveillance at the wildlife-livestock-human interface.

The majority of non-synonymous mutations identified in GD2411 were localized to the spike protein, particularly within regions associated with receptor binding and membrane fusion. These substitutions may modulate viral entry efficiency, immune evasion capacity, or cross-species transmissibility. Although fewer mutations were observed in RdRp and 3CLpro, these enzymes are essential for viral replication and proteolytic processing, respectively, and even single amino acid changes can significantly impact viral fitness. Notably, the S154A substitution in the nucleocapsid protein may influence viral RNA packaging or interactions with host cellular factors.

To further elucidate the functional implications of these variations, we conducted in silico structural analysis. For the RdRp protein, the mutation V3764I (located in the viral polymerase domain) involves a substitution between two hydrophobic amino acids, suggesting a conservative change that likely maintains the structural integrity of the catalytic core while potentially fine-tuning polymerase fidelity. However, the mutations observed in the Spike protein, particularly D489N and T512E, map to the surface-exposed loops of the S1 subunit. Structural modeling suggests that these substitutions alter the local electrostatic potential, which may influence receptor recognition or antigenicity, consistent with the high recombination rate observed in this region. These structural insights support the hypothesis that GD2411 is undergoing adaptive evolution. Functional studies are warranted to determine whether these mutations confer adaptive advantages.

Cross-species transmission events mediated by coronaviruses have occurred frequently (20–22). While BuCoV has not yet been associated with zoonotic transmission, its genetic plasticity, as demonstrated by frequent recombination and adaptive mutations, raises concerns regarding its potential to breach the species barrier. The white-eyed bulbul is a common urban and peri-urban bird in southern China, frequently interacting with human-modified environments. Should BuCoV acquire mutations facilitating mammalian cell entry—as observed in other delta coronaviruses—it could pose a spillover risk. Our findings highlight the necessity for expanded surveillance of avian delta coronaviruses, particularly in regions characterized by high biodiversity and anthropogenic pressure.

## Data Availability

The datasets presented in this study can be found in online repositories. The names of the repository/repositories and accession number(s) can be found below: https://www.ncbi.nlm.nih.gov/genbank/, PV544238.

## Supplementary material

SUPPLEMENTARY FIGURE S1 Geographic distribution of sampling sites and BuCoV prevalence in Guangdong Province, China. The map illustrates the 12 regions where a total of 2,145 fecal samples were collected. Sampling locations are indicated, with sample sizes provided for each site. BuCoV RNA was exclusively detected in Shenzhen (▲). No BuCoV positive samples were found in the other 11 regions.

SUPPLEMENTARY FIGURE S2 Melting curve analysis of BuCoV qPCR assay.Melting curve analysis showing a single specific peak at 80.8°C for BuCoV-positive samples (colored curves), confirming the specificity of the amplification. Negative controls (NTC) showed no primer-dimer formation or non-specific amplification products.

SUPPLEMENTARY FIGURE S3 Pairwise nucleotide identity matrices for BuCoV GD2411 and representative deltacoronaviruses. Figure A–E visualize sequence identities across 3CLpro,RdRp, Hel, S, and N genes among strains Dut148Cor1, Thr147Cor1, GD2411, HKU11-796, HKU11-700, and HKU11-934.

SUPPLEMENTARY FIGURE S4 Sequence logos showing amino acid variation at mutation sites in key viral proteins. (A-C) RdRp; (D) 3CLpro; (E) N protein; (F-H) S protein. The x-axis represents the position within each 20-amino acid window centered on the mutation site, and the y-axis indicates sequence conservation in bits. Letter height reflects the relative frequency of each amino acid at that position. Numbers above selected positions denote the corresponding residue numbers in the reference genome. Amino acids are color-coded by chemical properties: acidic (red), basic (blue), hydrophobic (black), neutral (gray), and polar (green).
