# Supplementary figures and images for: Molecular epidemiology and genomic analysis of bulbul coronavirus in Guangdong, China
Source: Front Vet Sci. 2026 Mar 4;13:1659863. doi: 10.3389/fvets.2026.1659863 (PMC12995750; doi:10.3389/fvets.2026.1659863)

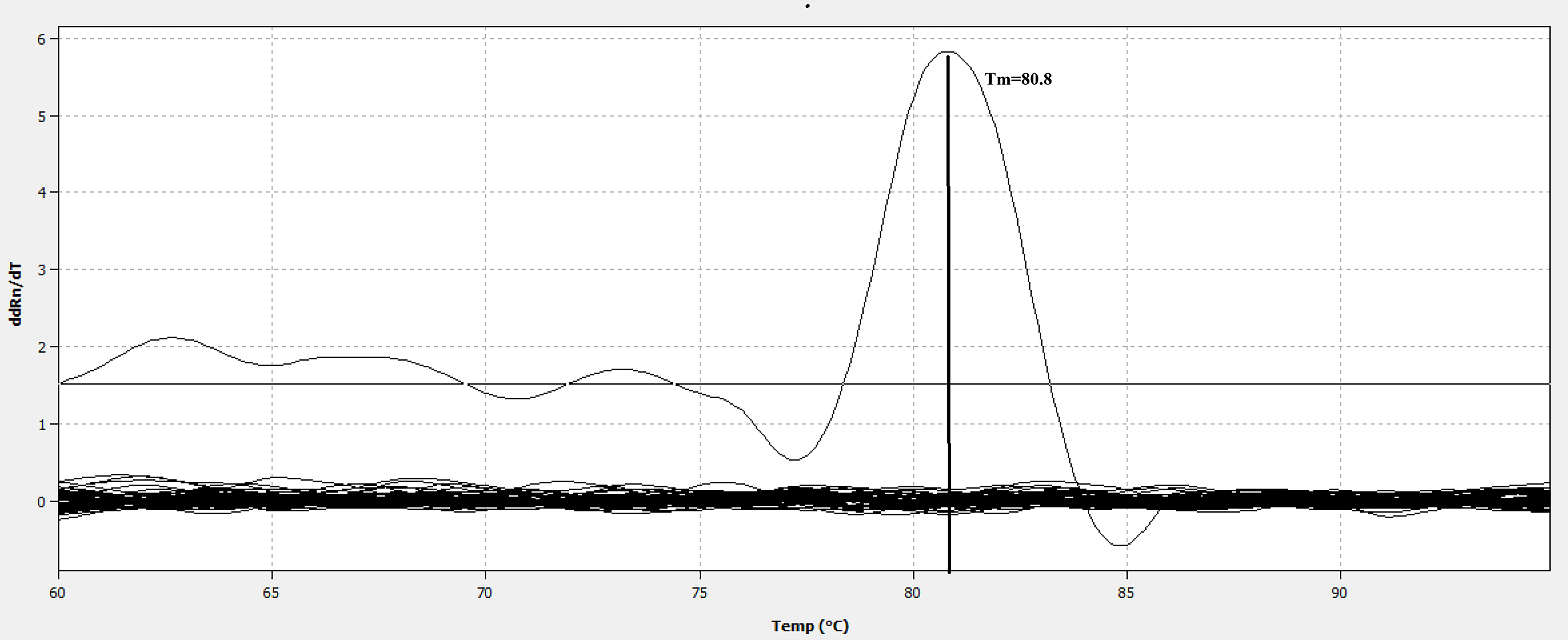

Supplement: SUPPLEMENTARY FIGURE S2 — Melting curve analysis of BuCoV qPCR assay.Melting curve analysis showing a single specific peak at 80.8°C for BuCoV-positive samples (colored curves), confirming the specificity of the amplification. Negative controls (NTC) showed no primer-dimer formation or non-specific amplification products. [file Image_2.tif]

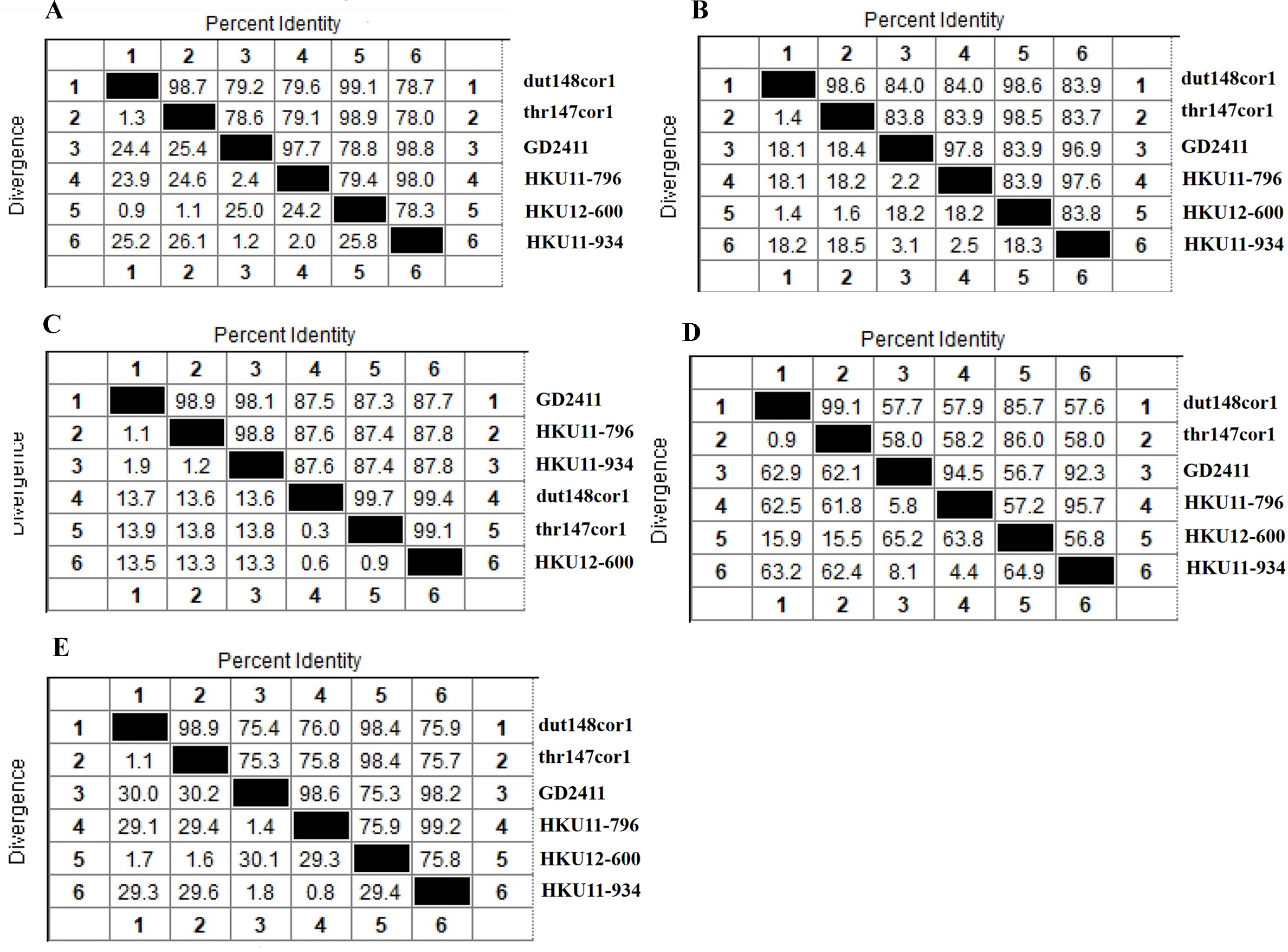

Supplement: SUPPLEMENTARY FIGURE S3 — Pairwise nucleotide identity matrices for BuCoV GD2411 and representative deltacoronaviruses. Figure A–E visualize sequence identities across 3CLpro,RdRp, Hel, S, and N genes among strains Dut148Cor1, Thr147Cor1, GD2411, HKU11-796, HKU11-700, and HKU11-934. [file Image_3.tif]

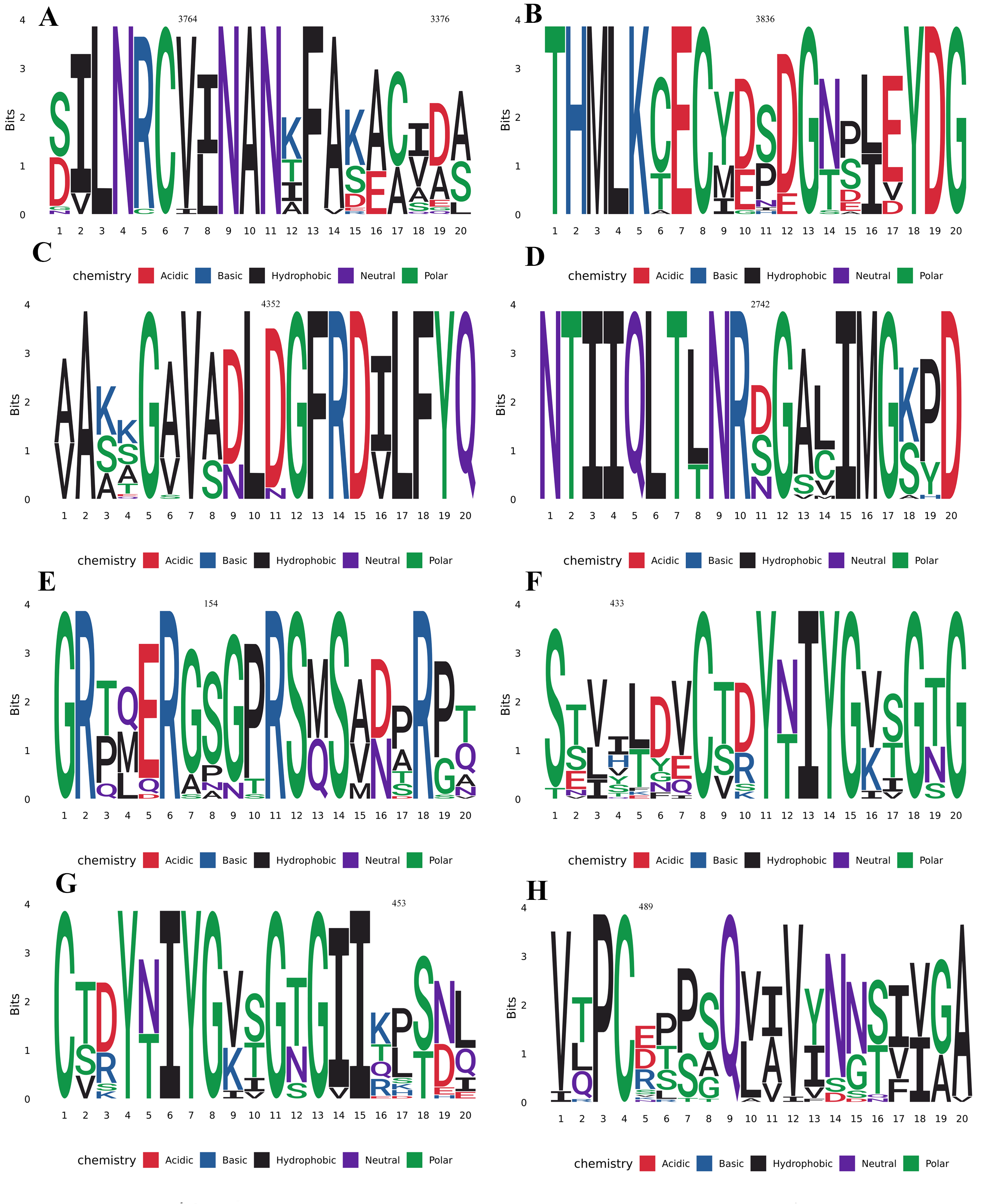

Supplement: SUPPLEMENTARY FIGURE S4 — Sequence logos showing amino acid variation at mutation sites in key viral proteins. (A-C) RdRp; (D) 3CLpro; (E) N protein; (F-H) S protein. The x-axis represents the position within each 20-amino acid window centered on the mutation site, and the y-axis indicates sequence conservation in bits. Letter height reflects the relative frequency of each amino acid at that position. Numbers above selected positions denote the corresponding residue numbers in the reference genome. Amino acids are color-coded by chemical properties: acidic (red), basic (blue), hydrophobic (black), neutral (gray), and polar (green). [file Image_4.tif]
